# Delayed diagnosis of coeliac disease increases cancer risk

**DOI:** 10.1186/1471-230X-7-8

**Published:** 2007-03-09

**Authors:** Marco Silano, Umberto Volta, Anna Maria Mecchia, Mariarita Dessì, Rita Di Benedetto, Massimo De Vincenzi, the collaborating centers of the Italian registry of the complications of coeliac disease

**Affiliations:** 1 Division of Food Science, Human Nutrition and Health, Istituto Superiore di Sanità, Viale Regina Elena 299, Rome, Italy; 2 Department of Internal Medicine, S. Orsola-Malpighi Hospital, Viale Massarenti 9, Bologna, Italy; 3 Department of Laboratory Medicine, University Hospital Tor Vergata, Viale Oxford 81, Rome, Italy

## Abstract

**Background:**

The association between coeliac disease (CD) and neoplasms has been long established, but few data are available about the risk factors. The aim of this paper is to estimate the risk of developing a neoplasm among non diagnosed coeliac patients and to evaluate if this risk correlates with the age of patients at diagnosis of coeliac disease.

**Methods:**

The study population consists of patients (n = 1968) diagnosed with CD at 20 Italian gastroenterology referral Centers between 1^st ^January 1982 and 31^st ^March 2005.

**Results:**

The SIR for all cancers resulted to be 1.3; 95% CI = 1.0–1.7 p < 0.001. The specific SIRs for non Hodgkin lymphoma was 4.7; 95% CI = 2.9–7.3 p < 0.001, for the small bowel carcinoma 25; 95% CI = 8.5–51.4 p < 0.001, for non Hodgkin lymphoma 10; 95% CI = 2.7–25 p = 0.01, finally for the stomach carcinoma 3; 95% CI = 1.3–4.9 p < 0.08. The mean age at diagnosis of CD of patients that developed sooner or later a neoplasm was 47,6 ± 10.2 years versus 28.6 ± 18.2 years of patients who did not.

**Conclusion:**

Coeliac patients have an increased risk of developing cancer in relation to the age of diagnosis of CD. This risk results higher for malignancies of the gastro-intestinal sites. An accurate screening for tumors should be performed in patients diagnosed with CD in adulthood and in advancing age.

## Background

Coeliac disease (CD) is a permanent autoimmune enteropathy triggered by the ingestion of gluten, the storage protein complex of wheat, rye and barley. Gluten causes, in genetically determined individuals carryng the HLA-DQ2/DQ8 haplotype, an inflammatory response of the small bowel mucosa, resulting in villous atrophy, infiltration of T-lymphocytes and hyperplasia of the cripts. The only known treatment for this disorder is the life – long withdrawn from the diet of the above mentioned cereals. CD occurs worldwide and its prevalence is estimated about 1/150 individuals [1-3].

The association between CD and neoplasms has been long established [4]. In the '60s, a population-based study has already reported a 100-fold increased risk of non-Hodgkin's lymphoma in patients affected by CD [5]. More recently, this risk has been resized to 3-fold by an Italian study and 9-fold by a study from States [6,7].

It has been also noted that CD patients have a higher risk of developing a small bowel adenocarcinoma respect to the general population and this neoplasm is the second invasive malignancy for incidence in these patients [7-10].

In contrast, several studies have shown a lower risk of breast cancer in patients affected by CD [11-13].

An increased mortality due to cancer in patients with CD has been also described [14,15]. There are considerable, but not definitive, evidences that the strict compliance to gluten-free diet is protective against the development of malignancies [16-18].

We carried out a perspective, population-based study on 1968 coeliac patients with the aim to evaluate the malignancy risk of developing a malignancy and to assess if the delayed diagnosis of coeliac disease and the consequent prolonged dietary exposure to gluten increase the risk of developing a neoplasm.

## Methods

### The Italian Registry of the complications of coeliac disease

The Italian Registry of the complications of coeliac disease was established at Istituto Superiore di Sanità, Rome in 1982. It is the largest database of coeliac patients in Italy. It contains the files of the coeliac patients referring to 20 Italian Gastroenterology Centers (collaborating Centers). The Istituto Superiore di Sanità, Rome, Italy provided a validate form in order to ensure uniformity in collecting data. The information about sex, date of birth, duration of symptoms before diagnosis and presence of malignancies were obtained upon the diagnosis of CD. All the filled forms were sent to Istituto Superiore di Sanità, where the data were entered in an electronic database (MS Access).

### Study population

The study population consists of the patients diagnosed with CD at Collaborating Centers between 1^st ^January 1982 and 31^st ^March 2005. All the patients were diagnosed with coeliac disease according to the National Institute of Health (NIH) criteria, including histological evidence of atrophy of small bowel mucosa and serological positivity for EmA and/or anti-tTg Ab (for the patients diagnosed after 1997) [19].

As our measure of relative risk we used overall and site-specific standardized incidence ratio (SIR) of cancer. We calculated the expected number of malignancies in each sex and 5-years stratum of our population by multiplying the number of patients in each stratum by site specific incidence rate from WHO Globoscan 2002 [20]. This database contains the annual incidence and prevalence rates of site specific malignancies in each World Countries according to age and sex.

### Statistical analysis

For the total cancers and for the most frequent localizations, the Standardized Incidence Ratio (SIR, ratio of the observed to the expected cases) was calculated, along with the 95% confidence interval, assuming a Poisson distribution of the population. We stratified the standardized morbidity ratio by the specific type of malignancy. The statistical analysis performed by SPSS software.

The collection of data have been approved by Istituto Superiore di Sanità (10959/8916), All the patients involved in the study signed the authorization for the handling of personal data and that the data were collected and stored according to national regulation.

## Results

### Population

One thousand nine hundred and sixty eight patients were enrolled. Out of them, 1485 were female, with a ratio female/male of 2.6. The mean age at diagnosis of CD was 36.2 ± 13.8. years, as shown in Table 1.

**Table 1:** Characteristics of 1968 patients with CD

**Characteristic**	**Number or Mean ± SD**
Female gender	1495
Mean age at diagnosis of CD	36.2 ± 13.8
Mean duration of symptoms before diagnosis of CD	6 ± 2.2

### Malignancy

Out of 1968 patients, 55 were diagnosed with a cancer (2.09%) before or simultaneously the diagnosis of CD versus 42.1 expected (SIR = 1.3; 95%CI = 1.0–1.7). Table 2 shows the malignancies observed and expected in our population. The most frequent malignancy resulted to be the gastro-intestinal non Hodgkin's lymphoma (n = 20), followed by colon carcinoma (n = 7), small bowel adenocarcinoma (n = 5), Hodgink's lymphoma (n = 4), stomach and breast carcinomas (n = 3). Other localizations included: liver, lung, ovary, thyroid cancer and mieloma (2 cases each) and acute leukemia, melanoma and uterus (1 cases each). No patient developed two or more cancers.

**Table 2:** Standardized Morbidity Ratio of the site specific malignancies developed before or simultaneously the diagnosis of CD

**Cancer**	**Observed**	**Expected**	**SIR(95%CI)**	**p**
**Cancer for whom CD patients showed a higher risk**				
Non Hodgkin's lymphoma	20	4.2	4.7 (2.9–7.3)	<0.001
Colon	7	6.2	1.1 (0.68–1.56)	<0.001
Small bowel	5	0.19	25 (8.5–51.4)	<0.001
Hodgkin's lymphoma	4	0.4	10 (2.7–25)	0.01
Stomach	3	1	3 (1.3–4.9)	0.08
**Cancer for whom CD patients showed a lower risk**				
Breast	3	14	0.2 (0.04–0.62)	<0.001
Others	13	12	1 (0.9–8.5)	0.06
**TOTAL**	**55**	**42.1**	**1.3 (1.0–1.7)**	**0.001**

Considering the SIR for site-specific malignancies, it resulted 4.7 for Non-Hodgkin's lymphoma, 26 for small bowel carcinoma, 3 for the stomach cancer and 10 for the Hodgkin's lymphoma. The risk for breast cancer resulted considerably lower than the general population.

The mean age at diagnosis of CD for the patients diagnosed with a cancer before o simultaneously was 47,6 ± 10.2 years, as shown in table 3. It was significantly higher than the age at diagnosis of CD of the patients who did not develop a malignancy (28.6 ± 18.2 years).

**Table 3:** Mean age at diagnosis of CD of patients diagnosed with cancer before or simultaneously compared with the mean age of diagnosis of malignancy in the general population

**Cancer**	**Mean age ± SD at diagnosis of CD**
Non Hodgkin's lymphoma	46.1 ± 13.8
Small bowel	59 ± 10
Colon	51.3 ± 9.4
Stomach	53.5 ± 15.4
Hodgkin's lymphoma	54.3 ± 6
Breast	41.2 ± 12.8
**All neoplasms**	**47.6 ± 10.2**

## Discussion

This study demonstrates that coeliac patients have an increased risk of developing cancer in relation to the age of diagnosis of CD. In fact, the mean age at diagnosis of CD in the group of patients that developed sooner or later a cancer is higher than that of patients who did not develop a cancer. Some Authors have supposed that the diagnostic delay is a risk factor for developing a malignancy because of the prolonged period of dietary exposure to gluten [15,16]. This risk is more relevant for the intestine -specific cancers such as small bowel carcinoma and non Hodking's lymphoma.

No cases of oropharyngeal and esophageal squamous carcinoma have been found, although Askling et al reported a high risk in coeliac population [12]. It is noteworthy that in the Swedish study, the patients were selected among those requiring hospitalization, so the severity of disease was a selection bias to be considered. In addition, there could be some genetic and environmental factors, such as the diet, exerting a protective effect towards tumors of the upper gastrointestinal tract among the Italian people. As matter of the fact, no excess of non hematological malignancies was found by a previous Italian population-based study [15].

Small bowel carcinoma is a rare malignancy that usually arises trough an adenoma-carcinoma sequence [21-23]. However, among our population, only a patient developed intestinal adenomas. These data did not support the theory that a premalignant lesion exists between villous atrophy and small intestine carcinoma, lending support to previous reports [24]. While small intestine carcinoma is a male predominant malignancy, four out of the five patients affected by this cancer were female. Since CD is prevalent among women, this finding is a further confirm of the link between small bowel neoplasm and CD.

From a public health perspective, the overall risk of developing a cancer in coeliac population found in this study and the uncertain protective effect of gluten free diet do not support the opportunity of a serological screening for coeliac disease on general population in order to prevent a malignancy. But the high mean age at the diagnosis of CD for the group of patients that developed a cancer suggests that an accurate search for malignancies should be performed in patients diagnosed with CD as adult and in the elderly.

This study presents some potential limitation; first of all, the ascertainment bias. This can only be addressed by a prospective study with age- and gender- matched controls. Moreover, all the Clinical Centers involved are tertiary centers for the diagnosis and treatment of gastrointestinal diseases. Then, the accurate and frequent clinical controls the patients with CD use to undergo to, can lead to the diagnosis of a higher cases of malignancies. So, an excess of the risk for tumors is likely to be estimated, particularly that of gastrointestinal sites, since they can be detected during the endoscopy usually performed for the hystological diagnosis of CD [25]. This work considered the incidence of malignancies before and/or simultaneously the diagnosis of CD only and not during the follow-up. Until the diagnosis of CD, the patients do not have more frequent clinical control than healthy population.

## Conclusion

This paper confirms that the gluten-free diet is likely to protect from the development of malignancies in CD patients, since higher is the age at diagnosis of CD, higher is the risk of developing a malignancy, Therefore, the importance of a prompt diagnosis of CD is emphasized. Our data require to be confirmed by larger population based studies, but some implications for an accurate screening for cancers in people with CD are added.

## Competing interests

The Author(s) declare that they have no competing interests.

## Authors' contributions

MS designed the study, organized the database, interpreted the data, drafted the manuscript and performed the statistical analysis. UV designed the study, revised critically the manuscript and added important points to the discussion, collected the data, performed the statistical analysis. AMM and RdB organized the electronic database and collected the data. MdV conceived the study, coordinated the collaborating centers and helped the draft of the manuscript. Collaborating Centers enrolled the patients, collected and interpreted the data. All Authors approved the final draft of the manuscript.

## Collaborating Centers

**Cattedra di Medicina Interna II, Univ. Cattolica, Roma**, G.Gasbarrini, M.D., V. De Vitis, M.D.; **Dipartimento di Pediatria, Univ. Federico II, Napoli**, L. Greco, M.D., S. Auricchio, M.D.; **Ospedale per gli infermi di Faenza – Azienda USL di Ravenna**, D.Santini, M.D., F. Scaggiante M., M.D., Vincenzi M., Federici M.D.; **Istituto Giannina Gaslini, Pediatria III, Genova**, E. Castellano, M.D., A.Calvi, M.D.; **Cattedra di Gastroenterologia, Dipartimento di Medicina Interna, Univ. Torino**, Sategna-Guidetti, S. Grosso; **Unità di Gastroenterologia, IRCCS Policlinico S. Matteo, Univ. Pavia**, Campanella J., M.D., Corazza G.R., M.D.; **Unita' Operativa di Nutrizione Clinica, Ospedale S. Eugenio, Roma**, G. Sandri, M.D., G. Giorgetti, M.D.; Monica Amici, **Dipartimento di Medicina Interna, Policlinico S. Orsola-Malpighi, Bologna**, U. Volta, M.D., L. De Franceschi, M.D.; **Servizio di Gastroenterologia ed Endoscopia Digestiva, Ospedale USL 9, Treviso**, S. Lo Perfido, M.D., ; **Divisione di Gastroenterologia, IRCCS Casa Sollievo Sofferenza, San Giovanni Rotondo, Foggia**, F. Perri, M.D., V. Festa M.D.,; **Clinica di Gastroenterologia ed Epatologia, Univ. Perugia**, M.A. Pelli, M.D., M.L. Cavalletti M.D.,; **Unità Operativa di Gastroenterologia ed Endoscopia Digestiva, Ospedale di Circolo e Fondazione Macchi, Varese**, S. Segato, M.D., M. Curzio M.D.,; **Servizio di Gastroenterologia ed Endoscopia Digestiva, Dipartimento Oncologia, Ospedale S. Giovanni AS, Torino**, M. Pennazio M.D.,, F.P. Rossini, M.D.; **Cattedra di Gastroenterologia, Ist. di Clinica Medica II, Univ. La Sapienza, Roma**, A. Picarelli M.D.; **Divisione di Gastroenterologia, Ospedale Mauriziano Umberto I, Torino**, A. Pera, M.D., E. Ercole M.D.; **Unità Operativa di Gastroenterologia, Dip.to di Patofisiologia Clinica, Univ. Pol. "Careggi" Firenze**, M.T. Passaleva; **Clinica Pediatrica, Servizio di Gastroenterologia, Univ. La Sapienza, Roma**, M. Barbato M.D.,; **Istituto di Medicina Interna, Univ. Cagliari**, P. Usai M.D.,, M.F. Dore M.D.,; **Divisione di Gastroenterologia, Ospedale Regionale, Bolzano**, F.Chilovi, M.D., L. Piazzi, M.D. L.Zancanella M.D., and **Servizio di Gastroenterologia Pediatrica e Servizio di Gastroenterologia, Univ. Modena**, V. Boarino, M.D., A. Ferrari M.D.,

## Pre-publication history

The pre-publication history for this paper can be accessed here:



## References

[B1] Fasano A, Catassi C (2001). Current approaches to diagnosis and treatment of celiac disease: an evolving spectrum. Gastroenterology.

[B2] Volta U, Bellentani S, Bianchi FB, Brandi G, De Franceschi L, Miglioli L, Granito A, Balli F, Tiribelli C (2001). High prevalence of celiac disease in Italian general population. Dig Dis Sci.

[B3] West J, Logan RF, Hill PG, Lloyd A, Lewis S, Hubbard R, Reader R, Holmes GK, Khaw KT (2003). Seroprevalence, correlates, and characteristics of undetected coeliac disease in England. Gut.

[B4] Fairly NH, Mackie FP (1937). The clinical and biochemical syndrome in lymphoma and allied diseases involving the mesenteric lymph glannds. BMJ.

[B5] Gough KR, Read AE, Naish IM (1962). Intestinal reticulosis as a complication of idiopatich steatorrhoea. Gut.

[B6] Catassi C, Fabiani E, Corrao G, Barbato M, De Renzo A, Carella AM, Gabrielli A, Leoni P, Carroccio A, Baldassarre M, Bertolani P, Caramaschi P, Sozzi M, Guariso G, Volta U, Corazza GR, Italian Working Group on Coeliac Disease and Non-Hodgkin's-Lymphoma (2002). Risk of Non-Hodgkin lymphoma in celiac disease. JAMA.

[B7] Green PH, Fleischauer AT, Bhagat G, et Goyal R, Jabri B, Neugut AI (2003). Risk of malignancy in patients with celiac disease. Am J Med.

[B8] Rampertab SD, Forde KA, Green PH (2003). Small bowel neoplasia in coeliac disease. Gut.

[B9] Howdle PD, Holmes GK (2004). Small bowel malignancy in coeliac disease. Gut.

[B10] Silano M, De Vincenzi M (2005). Small bowel malignancy at diagnosis of coeliac disease. Gut.

[B11] Swinson CM, Slavin G, Coles EC, Booth CC (1983). Coeliac disease and malignancy. Lancet.

[B12] Askling J, Linet M, Gridley G, Halstensen TS, Ekstrom K, Ekbom A (2002). Cancer incidence in a population-based cohort of individuals hospitalized with celiac disease or dermatitis herpetiformis. Gastroenterology.

[B13] Card TR, West J, Holmes GK (2004). Risk of malignancy in diagnosed coeliac disease: a 24-year prospective, population-based, cohort study. Aliment Pharmacol Ther.

[B14] Peters U, Askling J, Gridley G, Ekbom A, Linet M (2003). Causes of death in patients with celiac disease in a population-based Swedish cohort. Arch Intern Med.

[B15] Corrao G, Corazza GR, Bagnardi V, Brusco G, Ciacci C, Cottone M, Sategna Guidetti C, Usai P, Cesari P, Pelli MA, Loperfido S, Volta U, Calabro A, Certo M, Club del Tenue Study Group (2001). Mortality in patients with coeliac disease and their relatives: a cohort study. Lancet.

[B16] Holmes GK, Prior P, Lane MR, Pope D, Allan RN (1989). Malignancy in coeliac disease – effect of a gluten free diet. Gut.

[B17] Egan LJ, Walsh SV, Stevens FM, Connoly CE, Egan EL, McCarthy CF (1995). Celiac-associated lymphoma. A single institution experience of 30 cases in the combination chemotherapy era. J Clin Gastroenterol.

[B18] Lewis HM, Renaula TL, Garioch JJ, Leonard JN, Fry JS, Collin P, Evans D, Fry L (1996). Protective effect of gluten-free diet against development of lymphoma in dermatitis herpetiformis. Br J Dermatol.

[B19] National Institutes of Health (2005). Consensus Development Conference Statement on Celiac Disease. Gastroenterology.

[B20] Globocan 2002 database. http://www-dep.iarc.fr/globocan/database.htm.

[B21] Catassi C, Bearzi I, Holmes GK (2005). Association of celiac disease and intestinal lymphomas and other cancers. Gastroenterology.

[B22] Freeman HJ (2004). Lymphoproliferative and intestinal malignancies in 214 patients with biopsy-defined celiac disease. J Clin Gastroenterol.

[B23] Howdle PD, Jalal PK, Holmes GKT, Houlston RS (2003). Primary small bowel malignancy in the U.K. and its association with coeliac disease. QJM.

[B24] Bruno CJ, Batts KP, Ahlquist DA (1997). Evidence against flat dysplasia as a regional field defect in small bowel adenocarcinoma associated with celiac sprue. Mayo Clin Proc.

[B25] West J, Logan RF, Smith CJ, Hubbard RB, Card TR (2004). Malignancy and mortality in people with coeliac disease: population based cohort study. BMJ.

